# Influenza vaccination in patients with acute heart failure (PANDA II): study protocol for a hospital-based, parallel-group, cluster randomized controlled trial in China

**DOI:** 10.1186/s13063-024-08452-8

**Published:** 2024-11-25

**Authors:** Yiqun Zhang, Rong Liu, Yangyang Zhao, Zhiyan Wang, Chi Wang, Qiang Li, Dorothy Han, Craig S. Anderson, Xin Du, Jianzeng Dong

**Affiliations:** 1 Heart Health Research Center (HHRC), Beijing, China; 2 The George Institute for Global Health, UNSW, Sydney, Australia; 3 Institute for Science and Technology for Brain-Inspired Research, Fudan University, Shanghai, China; 4 Beijing Anzhen Hospital, the Capital Medical University, Beijing, China; 5 The First Affiliated Hospital of Zhengzhou University, Zhengzhou, Henan Province, China

**Keywords:** Influenza vaccine, Heart failure, Cluster randomized trial

## Abstract

**Background:**

Influenza vaccination confers broad benefits in the elderly and certain high-risk populations, but its effectiveness in patients with acute heart failure (HF) is uncertain. Rates of influenza vaccination are low in China due to poor awareness, cultural misunderstandings, and cost.

**Aims:**

To determine the effectiveness of influenza vaccination in patients with acute HF admitted to hospitals in China.

**Methods:**

The second Population Assessment of Influenza and Disease Activity (PANDA II) study is a two-arm, parallel-group, county-level hospital-based, cluster randomized controlled trial to determine the benefits and risks of full access to routine free influenza vaccination before hospital discharge, compared to routine limited use of influenza vaccination, on the primary endpoint of death or hospital readmission. Consecutive hospitalized patients at each site are enrolled to a target of 50 participants in each autumn–winter influenza outbreak period (October to March) over 3 consecutive years to reach the required sample size. Patients are centrally followed up at 1, 3, 6, and 12 months after hospital discharge (or death if earlier). Site numbers varied across year according to predicted influenza activity and logistical reasons.

**Conclusions:**

This study offers a unique chance to clarify uncertainties surrounding the effectiveness of influenza vaccination in patients with HF and to lay the groundwork for future prevention strategies.

**Trial registration:**

This trial was registered with the acronym PANDA II (Population Assessment of Influenza and Disease Activity) at ChiCTR.org.cn (ChiCTR2100053264). Registered on 17 November 2021.

**Supplementary Information:**

The online version contains supplementary material available at 10.1186/s13063-024-08452-8.

## Introduction

### Background and rationale {6a}

According to World Health Organization (WHO), influenza causes a severe illness in approximately 3 to 5 million people and results in 290,000 to 650,000 deaths each year [1]. Older people and those with cardiovascular (CV) disease are more likely to suffer a severe illness [2, 3]. Heart failure (HF) is a major clinical and public health concern that is estimated to affect 1.3% (13.7 million) of adult (age ≥ 35 years) population of China [4]. Patients who are hospitalized for HF have a high case fatality [5]. In Veterans Affairs Health Care System, the overall unadjusted rates of 1-year death or hospital readmission are 25.0% and 61.4%, respectively [6]. Up to one third of hospitalized cases of HF are triggered by respiratory infection [7], often from influenza [8], and this contributes to the poor outcome [9].

Influenza vaccination is a key management strategy in patients with CV disease [10]. In the Influenza Vaccination After Myocardial Infarction (IAMI) study involving 2532 patients, influenza vaccination produced a significant 28% relative reduction in the risk of the composite endpoint of all-cause death, myocardial infarction (MI), or stent thrombosis at 12 months [11]. Although guidelines and the World Health Organization recommend the use of influenza vaccination to improve survival after HF [12], the only randomized controlled trial (Influenza vaccine to prevent adverse vascular events [13]) failed to show a clear reduction in the primary endpoint of CV death, MI, and stroke, although there were 22% and 16% reductions in deaths and hospitalizations in the peak influenza seasons during follow-up. Influenza vaccination rates vary greatly across the world, from approximately 60% in patients with HF in Western Europe and the US [14], to less than 1% in China [15]. The low rate of vaccination in China can be explained by being an out-of-pocket expense [9], cultural beliefs, poor awareness [16], and safety concerns [14, 17].

The PANDA II study aims to provide evidence of the effectiveness of influenza vaccination for improving outcomes in patients with severe HF, using a hybrid effectiveness-implementation design. The feasibility in ensuring high levels of influenza vaccine coverage rates (VCR) in routine practice was confirmed in a pilot phase to the study in which 518 participants were recruited at 11 hospitals in the Henan Province of China between December 28, 2020, and January 29, 2021 [17].

### Objectives {7}

The objective is to determine whether the provision of free influenza vaccine before discharge from hospital has a beneficial effect on all-cause mortality or hospital readmission, compared to routine care, in patients with severe HF.

### Trial design {8}

**Fig. 1:**
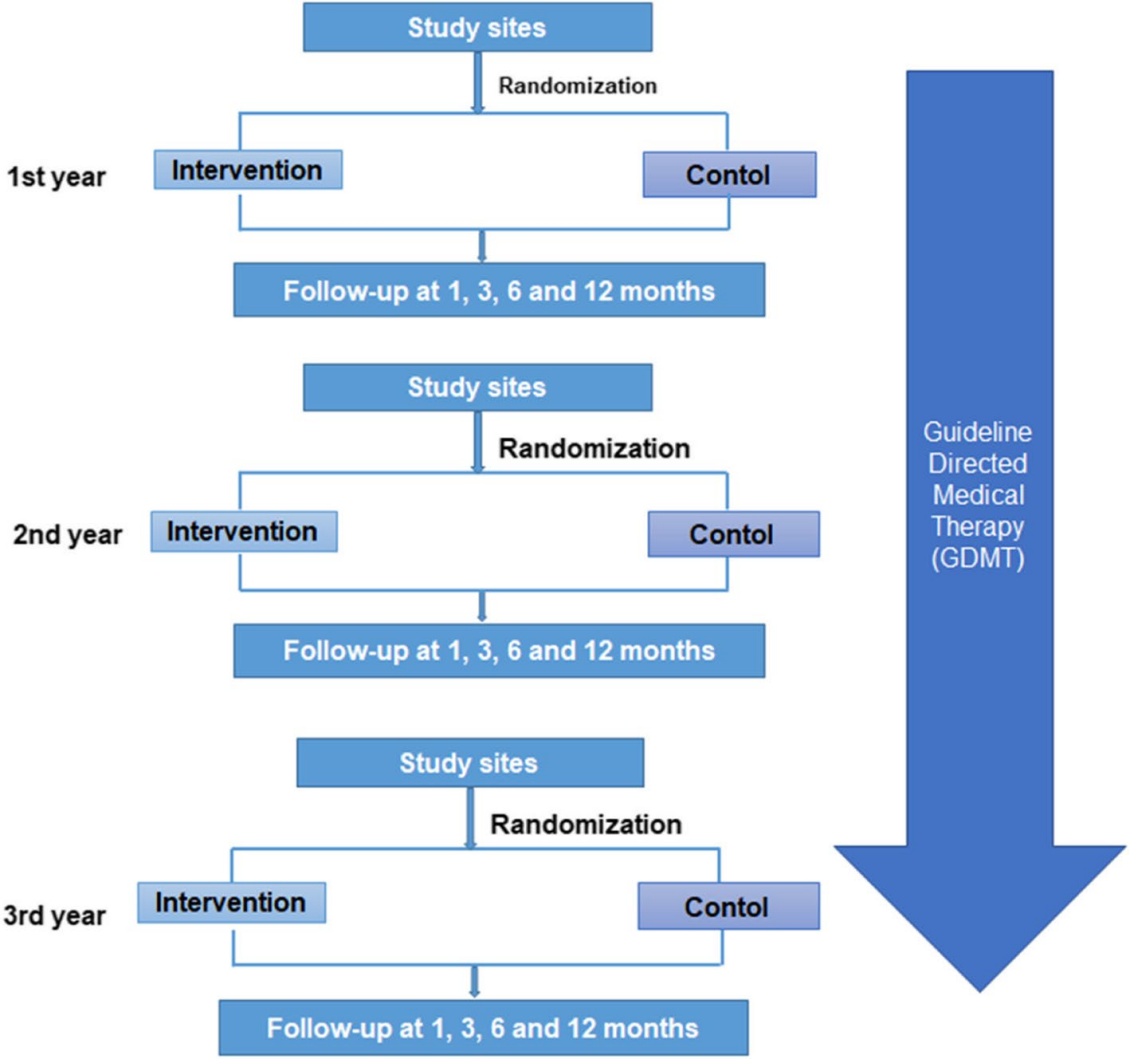
PANDA II main trail schema

This is a two-arm, parallel group, cluster-randomized 1:1 controlled trial designed to determine whether a program of free influenza vaccination in hospitalized HF patients improves outcome compared to usual standard of routine care. The unit of randomization is the hospital where investigators are required to enroll consecutive patients with HF during a defined winter period. Hospitals are centrally randomly allocated to be an intervention arm to provide a free influenza vaccination in wards devoted to the care of patients with HF. The control hospitals are to provide usual standard of care without routine Point of Vaccination (POV) but can make recommendations to patients to receive their influenza vaccination at community health care center. This crossover may reduce the study’s ability to detect the vaccination’s effect. To account for this, we estimated that 10% in the control group would receive the vaccine and adjusted the sample size accordingly. The study was planned to be undertaken in phases over three years and for participating hospitals to be re-randomized before each winter recruitment period. A detailed flowchart is shown in Fig. 1.

## Methods: participants, interventions, and outcomes

### Study setting {9}

The study is undertaken in county-level hospitals which serve relatively fixed local populations to avoid the risk of contamination. They are located in the geographically dispersed regions of the provinces of Henan, Jilin, Hebei, Shaanxi, Hunan, and Guangxi, to enhance the generalizability of the results (Appendix: participating hospitals). Up to 60 hospitals are randomly assigned to intervention or control groups in each year. At the completion of each recruitment period, an assessment is made of the recruitment volume, adherence to the protocol, and completeness of the data, to decide on their continued participation.

### Eligibility criteria {10}

#### Hospital-level inclusion criteria

Hospital sites are eligible if they meet all following requirement:

1. Ability to manage a high volume of patients with HF;
2. Capacity to enroll a target of 50 participants in a recruitment period;
3. Adequate levels of staff to participate in research;
4. Approval from the local government to provide influenza vaccination in the hospital.

#### Hospital-level exclusion criteria

Hospital sites are not eligible if they meet any of the following criteria:

1. Participation in another influenza vaccination-related program;
2. A policy of free influenza vaccination was already available for local residents.

#### Individual-level inclusion criteria

Patients are eligible if they meet all the following requirement:

1. Age ≥18 years;
2. Admission for hospital with a diagnosis of HF;
3. Severity of HF that is grade III or IV in the New York Heart Failure classification system;
4. Recruitment is to be consecutive to the quota of 50 participants;
5. Provision of written informed consent.

#### Individual-level exclusion criteria

Patients are not eligible if they meet any of the following criteria:

1. Previous enrollment in the study;
2. ≥ 2 admissions to hospital with HF in the past 2 months;
3. Known allergy to influenza vaccine;
4. Vaccination for COVID in the past 14 days;
5. Discharge from hospital with the intention of being transferred to another medical facility for further treatment.

## Who will take informed consent? {26a}

### Consent process for hospitals

Sites are required to consent patients prior to undertaking any study procedures including randomization. The process of consent has the approval of the General Manager or Head of the Cardiology Department.

### Consent process for individuals

Participants in the intervention arm provide consent for both vaccination and follow-up, whereas those in the control arm only provide consent for follow-up. Potential participants receive written information on the study and are given sufficient time to ask questions and make their decision. Refusal to participate does not influence the management of patients. Only patients with a signed informed consent are enrolled in the study. No study procedures or intervention will take place until the informed consent procedure is complete.

### Additional consent provisions for collection and use of participant data and biological specimens {26b}

This study does not involve any preservation or use of biological specimens.

## Interventions

### Intervention description {11a}

POV for influenza vaccination will be established in all study centers allocated to the intervention group. All participants receive vaccine of  the Sanofi Pasteur trivalent inactivated influenza vaccine (0.5 ml) or vaccine from another manufacturer with comparable efficacy within 24 h before discharge from hospital. The intervention involves education and training of the healthcare team in vaccination procedures. Patients eligible for the study who consent to follow-up will be enrolled regardless their wiliness to be vaccinated. Routine standard of care procedures is to be maintained for both groups. No intervention will be applied to the control arm but patients will be encouraged to seek influenza vaccination at a community health center or immunization clinic.

### Explanation for the choice of comparators {6b}

According to international guidelines and the WHO recommendation, high-risk populations received benefits from influenza vaccination if it is undertaken prior each influenza season. To ensure the application of ethical principles of equity and benefit, this study uses a hospital-based cluster design where participants allocated to control usual care arm are not denied the right to receive influenza vaccine. However, in previous reports [15] and our pilot phase [17], voluntary influenza vaccination rates were very low (< 1%) in China. Thus, the control arm is interpreted to be generally vaccination-free.

### Criteria for discontinuing or modifying allocated interventions {11b}

In the intervention group, only participants who provide consent to be vaccinated will receive influenza vaccine prior to hospital discharge. Participants who refuse the vaccine but consent to follow-up are still included in the intension-to-treat (ITT) analysis. The trial uses a one-off intervention, and no alternate or modified intervention will be implemented. However, participants have the right to discontinue their participation in the study at any time during follow-up, and an investigator has the right to modify receipt of the intervention if it is deemed in the best interests of the participant or guided by local policy.

### Strategies to improve adherence to interventions {11c}

A standardized educational curriculum on influenza vaccination in HF patients was developed for participating investigators, with the objective of ensuring that most participants are vaccinated in the intervention group. Moreover, study sites are required to provide screening logs to record the proportion of eligible patients who consent to participate. The study coordinating center will provide feedback to enhance adherence to study protocol.

### Relevant concomitant care permitted or prohibited during the trial {11d}

The treatment of HF is to follow the guidelines of the Chinese Society of Cardiology. There is no modification of background standard of care in both groups, except for the use of influenza vaccination in the intervention group.

### Provisions for post-trial care {30}

No post-trial care or compensation is anticipated.

### Outcomes {12}

The primary outcome is the composite of all-cause death or all-cause hospitalization in influenza season (defined by national CDC) within 1 year after hospital discharge. Hospitalization is defined as clinical manifestations that require admission (for at least 24 h) or emergency room stay (for at least 24 h). Non-influenza season events are unlikely to be attributable to influenza infections. Events beyond the corresponding influenza season are unlikely to be prevented by influenza vaccine. Influenza vaccines are estimated to have protective effects by generating antibodies within 30 days post-vaccination, so events occurring within 30 days after discharge are considered unlikely to be preventable by influenza vaccination. Thus, events that occur in that occur in the non-influenza season or within 30 days after hospital discharge will be excluded from primary outcome analysis.

The secondary outcomes are:

1. All-cause death within 1 year;
2. All-cause hospital re-admission within one year;
3. Composite of all-cause death or all-cause hospitalization within 6 months;
4. All-cause death within 6 months;
5. All-cause hospital re-admissions within 6 months.

Exploratory outcomes included the average number of all-cause hospital readmissions over a 1-year period, which refers to the average number of hospitalizations for any cause over a 1-year period. Another outcome is ILI, defined as ≥ 38 °C, cough or sore throat, within the past 7 days at each follow-up site. Safety outcomes are all-cause and cause specific serious adverse events (SAEs) according to standard definitions until the end of follow-up.

### Participant timeline {13}

The PANDA II study will be undertaken in an interrupted series over three 1-year periods. The time schedule of patient enrollment is between October 1, 2021, and March 31, 2022; October 1, 2022, and March 31, 2023; and October 1, 2023, and March 31, 2024. All patients are followed up over a 12-month period. Thus, the study is due for completion in April 2025. The overall timeline and the milestones are listed in Fig. 1, and the activities for each patient visit are presented in Appendix Table S1.

### Sample size {14}

The sample size for this study was based on the following assumptions:

1. A influenza infection rate of 15% in the background population
2. 80% of infected patient will experience the primary endpoint if infected with influenza
3. 90% of patients in the intervention group will receive the influenza vaccine, and 10% of patients in the control group received the vaccine
4. influenza vaccination has at least 60% protection for influenza infection
5. 10% of patients are lost to follow-up at 12 months

According to previous research, the 1-year death or hospital readmission rate for patients with HF is 50 per 100 person-years, after events in the non-influenza season or within 30 days of hospital discharge are excluded [18]. The intra-class correlation coefficient (ICC) for 1-year mortality or HF-specific readmission rates at participating hospitals was 0.03. A sample size of 122 hospitals participating over 3 influenza seasons in which 50 patients are recruited per hospital each season would provide 90% power at a two-tailed significance level of < 0.05. Sample sizes were calculated using PASS 2020 software under the “test for two proportions in a cluster randomized design” module.

### Recruitment {15}

Before randomization, investigators will screen potential hospitals through the national clinical research network. All eligible hospitals are first surveyed with a feasibility questionnaire to determine their willingness and capability to participate. Every eligible HF inpatient admitted to a study hospital is screened and invited to participate in the trial. The admission list and patient log from each participating site will be monitored to ensure sufficient consecutive HF patients are enrolled.

## Assignment of interventions: allocation

### Sequence generation, concealment, and implementation {16a,16b,16c}

Computer-generated random numbers are used to assign participating hospitals to the intervention or control group in a 1:1 ratio. Randomization is stratified by province to ensure the numbers of intervention and control hospitals are balanced.

Given the characteristic of the intervention, it will be impossible for the study investigator or the physicians to be blinded to the group allocation. The assignment of either intervention or control will be communicated to site investigators. However, the investigators at each site are blind to the sequence generation, thus ensuring that they have no opportunity to anticipate the allocation of their site until the study group assignment is revealed. In the second and the third influenza season, participating hospitals are re-randomized using the same process before the planned recruitment window.

## Assignment of interventions: blinding

### Who will be blinded {17a}

Given the nature of the study design, both study participants and site researchers will not be blinded. The data assessors and data analysts from coordinating center will be blind to the assignment of participants.

### Procedure for unblinding if needed {17b}

Not applicable.

## Data collection and management

### Plans for assessment and collection of outcomes {18a}

Data collection for both groups will be conducted concurrently and in parallel. Screening and baseline data will be collected and documented at the time of admission and during hospitalization by the pre-specified research team at each site. The population descriptive information, including demographics, anthropocentric, medical history, and concomitant medications, is collected by self-report. Results of laboratory test including blood, urine sampling, and echocardiogram will be retrieved. The electronic health records will be searched for source data validation, if available. Patients will be surveyed with quality of life and mood questionnaires: EQ-5D [19] and PHQ-9 [20]. Influenza vaccination data will be obtained prior to discharge. Centralized follow-up will be conducted by the coordinator center at each follow-up period to obtain information on deaths, re-hospitalization, occurrence of ILI, and any SAEs, along with the use of specific HF medications.

### Plans to promote participant retention and complete follow-up {18b}

Participants are required to provide four telephone numbers or contact details. If a participant is unable to complete a telephone follow-up, staff at the coordinator center will attempt to contact them through other available sources of information. If they are still unable to be assessed, their outcome or survival status will be confirmed through routine social network or health insurance systems, if available.

### Data management {19}

Data collection will be processed though customized web-based database. An electronic data capture system with range and logic checks will be used for both data entry and storage purposes. After the completion of data collection from various sources, the clinic coordinators will review each filled form for errors and completeness. As part of quality control measures, data reviews will be carried out to check consistency and logic issues on key variables. Data review reports will be generated and provided to sites and study committees on a regular basis to promote data quality.

### Confidentiality {27}

The research team has responsibility to ensure security and confidentiality of data at sites and the coordinating center. All individual and relevant site information will be de-identified before entry into the database and any statistical analysis to protect the privacy of all participants. Sources files or documents will be stored in locked filing cabinets at each site. Uploaded copies will be stored online in a non-URL accessible area that controlled by a system of user identification names and passwords encrypted with SSL encryption. Each site investigator must complete a data handling training program before they are given assess rights to the database. The study is conducted in compliance with Chinese national privacy and confidentiality laws.

### Plans for collection, laboratory evaluation, and storage of biological specimens for genetic or molecular analysis in this trial/future use {33}

No biological specimens will be involved in this study.

## Statistical methods

### Statistical methods for primary and secondary outcomes {20a}

#### General consideration

Continuous variables will be summarized by means (standard errors) and medians (inter-quartile intervals); if skewed, a transformation will be applied to induce approximate normality before testing. In any event that a suitable transformation cannot be found, a Wilcoxon test will be used. Descriptive statistics will be provided for patient characteristic, hospital characteristics, and safety data.

#### Endpoint analysis

The aim of the primary analysis will be to establish whether there are benefits in all-cause mortality or all-cause hospital readmission within 1 year (excluding events within 30 days post-discharge or occurring during a non-influenza season) from patients enrolled in the intervention hospitals compared with patients enrolled in the control hospitals. Aggregate cluster-level mean rates of primary endpoints between intervention clusters and control clusters will be compared by Wilcoxon rank-sum test. The intention-to-treat (ITT) principle will be applied.

In the secondary analysis, generalized linear mixed model will be performed to allow for the clustered nature of the data and to adjust the cluster-level covariates including province, hospital, and phase.

#### Interim analyses {21b}

No formal interim analysis will be conducted during the study. During the period of patient enrollment, interim analysis of major endpoints (including SAEs believed to be due to intervention) will be supplied by the independent trial statistician as soon as possible after the completion of patient follow-up during each influenza season, who will present these reports to the Chairman of the Data and Safety Monitoring Board (DSMB) along with any other analyses that the committee may request. The DSMB will advise the chairman of the steering committee if, in their view, the randomized comparisons in the trial have provided both (i) “proof beyond reasonable doubt” that one particular intervention is clearly indicated or clearly contraindicated in terms of a net difference in outcome and (ii) evidence that intervention expected to influence the patient management. Appropriate criteria of proof beyond reasonable doubt cannot be pre-specified precisely, but a difference of at least 3 standard deviations using the Haybittle-Peto stopping rule in an interim analysis of a major endpoint may be needed to justify halting or modifying the study prematurely. Site principal investigators (PIs) and study and steering committee personnel will remain blinded to the results of the interim analyses and safety assessments.

#### Methods for additional analyses {20b}

Subgroup analyses will be performed by province, year, and severity of HF (NYHA classification). For the degree of influenza activity, it is planned to classify influenza activity using the median ILI + /ILI of North and South China during the study period.

#### Methods in analysis to handle protocol non-adherence and any statistical methods to handle missing data {20c}

Consistent with an ITT analysis, this study will involve all participants in the primary analyses regardless of adherence. For participants lost to follow-up, all available information until the time of death or loss to follow-up will be used in analysis [21]. Missing values will be identified and replaced by a random sample of plausible values imputations (completed datasets).

## Oversight and monitoring

### Composition of the coordinating center and trial steering committee {5d}

The Heart Health Research Center (HHRC) in Beijing, China, coordinates the study and manages its daily operations. Responsibilities include center enrollment, ethics applications for each participating site, training local investigators to ensure high-quality trials, influenza vaccine procurement and distribution, data monitoring, and organizing meetings with the steering committee, DSMB, and investigators.

The steering committee, composed of trialists, cardiologists, and vaccine experts, is responsible for approving the study protocol and any amendments. The committee oversees the trial’s quality and progress and reviews DSMB reports to determine whether to continue or halt the trial.

This study plan to involve a comprehensive approach to data monitoring, combining central (remote) and on-site supervision. Prior to the initiation of the study at each participating site, all designated research personnel, including the PI, co-investigators, and research nurses/coordinators, receive comprehensive training on study procedures from the operational staff. Regular remote monitoring of data completion and quality is conducted throughout the study. Furthermore, the sponsor or their delegates will conduct annual visits to each participating center, in accordance with the study monitoring plan, to ensure adherence to the protocol, ICH-GCP guidelines, and relevant ethical and regulatory requirements. The monitor will meticulously verify participant informed consent and eligibility, and review relevant source documents, in accordance with a detailed monitoring plan. Upon completion of the study, the investigator is responsible to ensure the implementation of long-term storage plans for all relevant data and source documentation.

### Composition of the data monitoring committee, its role and reporting structure {21a}

An independent DSMB will conduct a comprehensive review of the study’s safety and outcomes every 6 months. The DSMB’s responsibilities encompass monitoring blinded response variables as well as SAEs to promptly identify any potential beneficial or detrimental effects for safety surveillance purposes. The DSMB will be governed by a charter that comprehensively delineates their responsibilities, procedures, and commitment to confidentiality. They will diligently furnish reports to the Central Coordinating Center (CCC) regarding recommendations on whether to continue or temporarily suspend recruitment for the study, based upon review of unblinded data from each influenza season’s conclusion. Additionally, they will monitor vaccination rate, influenza activity levels, vaccine strain compatibility with circulating strains, and attrition rates among participants as well as event occurrence frequencies. Safety reports will be made by the independent statistician after the occurrence of every 5 suspected unexpected serious adverse reaction (SUSAR) or after the occurrence of every 10 SAEs. Based on the outcomes of prior analyses, the DSMB may suggest to the steering committee a potential relaxation of these criteria.

### Adverse event reporting and harms {22}

The study vaccination’s safety will be assessed using a registry. Interim analyses on SAEs related to the intervention will be provided by an independent statistician to the DSMB Chair during each influenza season. If there is a significant difference (3 standard deviations) in a major endpoint based on Haybittle-Peto stopping rule, the study may be halted or modified. Adverse event following immunization (AEFI) will be reported to the steering committee and manufacturer’s pharmacovigilance team. SAEs must be reported to CCC within 24 h of the study team’s awareness, using the eCRF. Site investigators must also report SAEs to the relevant ethics committee within their specified timeframe.

All SAEs, whether related to influenza vaccine or not, must be evaluated for unexpectedness and vaccine association. Endpoints defined in the study will not be reported as adverse events. However, any other clinical signs and symptoms that are unexpected will be reported within 7 days post-vaccination.

### Frequency and plans for auditing trial conduct {23}

The Project Management Group will meet weekly to review the trial’s conduct. An independent DSMB will meet every 6 months to assess participant safety, study conduct, and progress. The DSMB will then provide recommendations to the steering committee regarding the continuation, modification, or termination of the trial.

The study may also undergo external audits conducted by third-party entities and inspections carried out by government regulatory authorities. Access to source documents, original CRF, eCRFs, and other relevant files must be readily available at all participating sites for the purpose of monitoring and auditing, as per mutually agreed upon schedules throughout the duration of the study and following its completion.

### Plans for communicating important protocol amendments to relevant parties (e.g., trial participants, ethical committees) {25}

Approved protocol amendments will first be communicated to sponsors and then in writing to funders. Revised protocols and related documents will be submitted to the ethics committee for records. Site investigators and their respective ethics committees will be notified of these amendments. If a site ethics committee requires additional review, a new amendment must be submitted to that center. The site committee’s decision should be reported to and recorded by the central ethics committee and the coordination center (HHRC) and noted in the site investigator’s file. If no further review is needed, the revised protocol should only be archived in the site file. Protocol modifications, project status, and study information will be updated on https://www.chictr.org.cn.

In cases when the amendment is required to protect the subject safety, the amendment can be implemented prior to ethics committee approval. Subject safety takes precedence over protocol adherence. In such cases, immediate actions are reported to the HHRC and the ethics committee within 72 h.

Deviations or violations will be documented using the Protocol Deviation/Violation Form, and data from monitoring activities will be collected and communicated with sites. Site-specific variations require steering committee ratification.

### Dissemination plans {31a,c}

Publication plans and dissemination materials will be discussed within the steering committee. Data sharing arrangement will be discussed on a case-by-case basis with the PI. Publication of the main reports from the study will be in the name of the “PANDA II Study Collaborative Investigators” with credit assigned to the collaborating investigators and other research staff. Full editorial control will reside with a writing committee approved by the steering committee.

As this is a multicenter study, investigators can publish or present their own site’s results with steering committee permission after the main result is published. They must give the steering committee 30-day notice and provide copies of any study result reports for review. The steering committee shall have the right to review and comment as regards the accuracy of the information and the protection of the rights, ensuring a fair and balance representation, in compliance with regulations.

### Patient public involvement

In the design of the protocol, a pilot study using mixed methods was conducted from December 2020 to April 2021 across 11 hospitals, involving interviews with 51 key informants (patients, health professionals, policymakers). This study aimed to assess the feasibility, acceptability, barriers, and facilitators of the intervention to guide modifications before the large-scale trial [22].

## Discussion

The PANDA II study aims to provide direct and reliable evidence on the effectiveness and safety of influenza vaccination in patients with acute HF. It follows the implementation strategy proposed in a pilot study [17], where a 90% vaccination coverage rate (VCR) was achieved in the intervention group, and remained as low as < 1% in the control group as influenza vaccines are not covered by the national immunization program in regions of China [16]. A strength of this trial is the design, which provides an exceptional opportunity to evaluate both efficacy and policy perspectives regarding the protective effect of influenza vaccination in patients with HF.

Another strength of this trial lies in its recruitment strategy, which leverages the existing HF health provider network and ensures the inclusion of consecutive and representative participants. The construction of POV in hospitals during the trial enhances the intervention implementation and site adherence, which can be scaled up into a routine practice should the intervention prove to be effective. Despite encountering logistical challenges associated with the conduct of such a large cluster trial in a range of low resource settings where there are varying levels of trial experience, our research team has implemented centralized monitoring and tracking systems for participating hospitals and patients to ensure data quality. Furthermore, the study faced difficulties due to the COVID-19 pandemic. It can be expected that the outcome event rates will differ according to times of co-circulation the COVID-19 virus and influenza activity was low in the period of quarantine policy in 2021. We did not initiate the study until the influenza activity surveillance reported an ILI + /ILI > 3% at week 38 of 2021. In 2022, a higher mortality rate in patients with HF was observed due to COVID-19 infection, which may dilute the effect of influenza vaccination. However, stratified cluster randomization design is expected to keep the influence balanced between study arms. A sensitivity analysis will be performed to evaluate the impact of COVID-19.

Our study is a well-powered and on schedule to resolve uncertainty over influenza vaccination in an effectiveness-implementation hybrid design, while also providing a model for future preventive strategies in patients. Insights gained from this study will inform and enhance HF management practices in regions where influenza vaccine uptake remains low.

## Trial status

The study has received approval from relevant ethics committees and regulatory bodies at both national and local levels in China. Patient enrollment began on December 1, 2021, and concluded on February 8, 2024. The current protocol is version 3.0 (date: April 30, 2024), and all protocol amendments have been approved by study steering committee and Medical Ethics Committee of Beijing Anzhen Hospital affiliated to Capital Medical University (Research Ethics Review (2021) No.18) and communicated with investigators and DSMB members.

## Supplementary Information

Supplementary Material 1. Appendix Table S1.

Supplementary Material 2. SPIRIT Checklist for Trials.

Supplementary Material 3.

## Data Availability

As a multi-center experiment, the full analysis set is only available to the lead researcher team. The investigator has access to the data set of his sub-center but not to the full analysis set. A password-protected file site will contain the clean data set without any participant identification information for confidentiality purpose. The data will only be made available upon reasonable request and with the approval of the PI.
