# Correction: Influenza vaccination in patients with acute heart failure (PANDA II): study protocol for a hospital-based, parallel-group, cluster randomized controlled trial in China

**DOI:** 10.1186/s13063-024-08686-6

**Published:** 2024-12-11

**Authors:** Yiqun Zhang, Rong Liu, Yangyang Zhao, Zhiyan Wang, Chi Wang, Qiang Li, Dorothy Han, Craig S. Anderson, Xin Du, Jianzeng Dong

**Affiliations:** 1 Heart Health Research Center (HHRC), Beijing, China; 2 The George Institute for Global Health, UNSW, Sydney, Australia; 3 Institute for Science and Technology for Brain-Inspired Research, Fudan University, Shanghai, China; 4 Beijing Anzhen Hospital, the Capital Medical University, Beijing, China; 5 The First Affiliated Hospital of Zhengzhou University, Zhengzhou, Henan Province China

**Correction: Trials 25, 792 (2024)**

**https://doi.org/ 10.1186/s13063-024-08452-8**

Following the publication of the original article [1], we were notified of a few errors in the affiliation numbers assigned to the 4th and last author, as well as in the first and last corresponding email addresses (changes marked in bold).

- Originally assigned affiliations:
  Yiqun Zhang1, Rong Liu2, Yangyang Zhao1, Zhiyan Wang**3,4**, Chi Wang1, Qiang Li2, Dorothy Han1, Craig S. Anderson1,2,3*, Xin Du1,4* and Jianzeng Dong3,5*
- Correct affiliation assignment:
  Yiqun Zhang1, Rong Liu2, Yangyang Zhao1, Zhiyan Wang**4**, Chi Wang1, Qiang Li2, Dorothy Han1, Craig S. Anderson1,2,3*, Xin Du1,4* and Jianzeng Dong4,5*
- Originally published correspondence emails: Canderson@georegeinstitute.org, duxinheart@sina.com, jz_dong@163.com
- Corrected correspondence emails: canderson@georgeinstitute.org.**au**, duxinheart@sina.com, jz_dong@**126**.com

In addition, on page 2, in the Trials design {8} section, the sentence *“Hospitals are centrally randomly allocated to be an intervention arm to provide a free POV influenza vaccination in wards devoted to the care of patients with HF.”* needs to read *“Hospitals are centrally randomly allocated to be an intervention arm to provide a free influenza vaccination in wards devoted to the care of patients with HF.”* AND sentence “*The control hospitals are to provide usual standard of care without routine POV but can make recommendations to patients to receive their influenza vaccination at community health care center.”* should read *“The control hospitals are to provide usual standard of care without routine Point of Vaccination (POV) but can make recommendations to patients to receive their influenza vaccination at community health care center.”* (the word **POV** has been removed from both sentences).

On page 8, under the Plans for communicating important protocol amendments to relevant parties (e.g., trial participants, ethical committees) {25} section, ClinicalTrials.gov. was changed into https://www.chictr.org.cn.

The original article has been corrected.
