# Supplementary material for: Influenza vaccination in patients with acute heart failure (PANDA II): study protocol for a hospital-based, parallel-group, cluster randomized controlled trial in China
Source: Trials. 2024 Nov 25;25:792. doi: 10.1186/s13063-024-08452-8 (PMC11587674; doi:10.1186/s13063-024-08452-8)
Supplement: Supplementary file 1 — Supplementary Material 1. Appendix Table S1. [file 13063_2024_8452_MOESM1_ESM.doc]

**Table 1. Table of activities**

| **Study Period** | **A**  **Baseline**  **Assessment** | **B**  **Discharge and Intervention** | **C**  **Follow up at 1 month** | **D**  **Follow up at 3 month** | **E**  **Follow up at 6 month** | **F**  **Follow up at 12 month** |
| --- | --- | --- | --- | --- | --- | --- |
| **TIMEPOINT** | **0 week** | **1 month** | **2 months** | **3 months** | **6 months** | **12 months** |
| **Window for evaluation** | **N/A** | **±10 days** | **±10 days** | **±10 days** | **±30 days** | **±30 days** |
| **ENROLMENT** |  |  |  |  |  |  |
| **Eligibility screen** | **X** |  |  |  |  |  |
| **Informed consent** | **X** |  |  |  |  |  |
| **Allocation** | **X** |  |  |  |  |  |
| **INTERVENTIONS** |  |  |  |  |  |  |
| Trivalent inactivated influenza vaccine (0.5 ml) |  | **X** |  |  |  |  |
| Voluntary influenza vaccination |  | **X** |  |  |  |  |
| **ASSESSMENTS** |  |  |  |  |  |  |
| **Laboratory examination** |  |  |  |  |  |  |
| Complete blood count | **X** |  |  |  |  |  |
| C-reactive protein | **X** |  |  |  |  |  |
| Albumin | **X** |  |  |  |  |  |
| Renal and Liver Function and electrolytes | **X** |  |  |  |  |  |
| Blood Lipids | **X** |  |  |  |  |  |
| Blood Fasting glucose, HbA1C | **X** |  |  |  |  |  |
| BNP or NT-proBNP | **X** |  |  |  |  |  |
| TnI | **X** |  |  |  |  |  |
| INR | **X** |  |  |  |  |  |
| **Clinical examination** |  |  |  |  |  |  |
| Blood Pressure and Heart Rates | **X** |  |  |  |  |  |
| Height and Weight | **X** |  |  |  |  |  |
| Weight monitoring during hospitalization | **X** |  |  |  |  |  |
| UCG | **X** |  |  |  |  |  |
| ECG | **X** |  |  |  |  |  |
| **Questionnaires** |  |  |  |  |  |  |
| Demographics data | **X** |  |  |  |  |  |
| Medical History | **X** |  |  |  |  |  |
| Family History | **X** |  |  |  |  |  |
| Lifestyle-Smoking, Alcohol usage | **X** |  |  |  |  |  |
| HF medications and adherence | **X** | **X** | **X** | **X** | **X** | **X** |
| Interventional surgery | **X** | **X** | **X** | **X** | **X** | **X** |
| Concomitant Medications | **X** | **X** | **X** | **X** | **X** | **X** |
| Adverse Events | **X** | **X** | **X** | **X** | **X** | **X** |
| Health related quality of life |  |  |  |  |  |  |
| EQ-5D | **X** |  |  |  |  |  |
| PHQ-9 |  | **X** | **X** | **X** | **X** | **X** |
| **Outcome Ascertainment** |  | **X** | **X** | **X** | **X** | **X** |
