# Supplementary material for: Influenza vaccination in patients with acute heart failure (PANDA II): study protocol for a hospital-based, parallel-group, cluster randomized controlled trial in China
Source: Trials. 2024 Nov 25;25:792. doi: 10.1186/s13063-024-08452-8 (PMC11587674; doi:10.1186/s13063-024-08452-8)
Supplement: Supplementary file 3 — Supplementary Material 3. [file 13063_2024_8452_MOESM3_ESM.docx]

List of participating 93 hospitals.

| **Serial number** | **province** | **Center Name** |
| --- | --- | --- |
| 1 | Anhui Province | Dingyuan County General Hospital, Anhui Province |
| 2 |  | Anhui Funan County Traditional Chinese Medicine Hospital |
| 3 |  | The First People's Hospital of Huoqiu County, Anhui Province |
| 4 | Guangxi Zhuang Autonomous Region | Yongfu County People's Hospital, Guangxi Zhuang Autonomous Region |
| 5 | Hebei Province | Anguo City Hospital, Hebei Province |
| 6 |  | Hebei Dachang Hui Autonomous County People's Hospital |
| 7 |  | Dacheng County Hospital, Hebei Province |
| 8 |  | The Seventh People's Hospital of Hebei Province |
| 9 |  | Gaoyang County Hospital, Hebei Province |
| 10 |  | People's Hospital of Gu'an County, Hebei Province |
| 11 |  | The Fourth People's Hospital of Langfang City, Hebei Province |
| 12 |  | Langfang People's Hospital, Hebei Province |
| 13 |  | Nanpi County People's Hospital, Hebei Province |
| 14 |  | Xianghe County People's Hospital, Hebei Province |
| 15 |  | Xianghe County Traditional Chinese Medicine Hospital, Hebei Province |
| 16 |  | Yutian County Hospital, Hebei Province |
| 17 | Henan Province | Dancheng County People's Hospital, Henan Province |
| 18 |  | Dengfeng People's Hospital, Henan Province |
| 19 |  | Dengzhou People's Hospital, Henan Province |
| 20 |  | Fengqiu County People's Hospital, Henan Province |
| 21 |  | Gongyi People's Hospital, Henan Province |
| 22 |  | Gushi County Traditional Chinese Medicine Hospital, Henan Province |
| 23 |  | Guangshan County People's Hospital, Henan Province |
| 24 |  | Huaxian People's Hospital, Henan Province |
| 25 |  | Huaibin County People's Hospital, Henan Province |
| 26 |  | People's Hospital of Jiyuan City, Henan Province |
| 27 |  | Jiaxian People's Hospital, Henan Province |
| 28 |  | People's Hospital of Linzhou City, Henan Province |
| 29 |  | The First People's Hospital of Lingbao City, Henan Province |
| 30 |  | Lushan County People's Hospital, Henan Province |
| 31 |  | Luanchuan County People's Hospital, Henan Province |
| 32 |  | The Second People's Hospital of Mengjin District, Henan Province |
| 33 |  | People's Hospital of Nanzhao County, Henan Province |
| 34 |  | People's Hospital of Ningling County, Henan Province |
| 35 |  | People's Hospital of Qinyang City, Henan Province |
| 36 |  | Ruyang County People's Hospital, Henan Province |
| 37 |  | The First People's Hospital of Ruzhou City, Henan Province |
| 38 |  | Henan Shangcheng County People's Hospital |
| 39 |  | Henan Shangshui County People's Hospital |
| 40 |  | Weishi County People's Hospital, Henan Province |
| 41 |  | Wen County People's Hospital, Henan Province |
| 42 |  | Xixian People's Hospital, Henan Province |
| 43 |  | Yexian People's Hospital, Henan Province |
| 44 |  | Yongcheng People's Hospital, Henan Province |
| 45 |  | Yuanyang County Central Hospital, Henan Province |
| 46 |  | Changge People's Hospital, Henan Province |
| 47 |  | Zhecheng County People's Hospital, Henan Province |
| 48 | Heilongjiang Province | Baoquanling Branch Central Hospital of Heilongjiang Province |
| 49 |  | Heilongjiang Beidahuang Group builds Sanjiang Hospital |
| 50 |  | Bin County People's Hospital, Heilongjiang Province |
| 51 |  | Fuyu County People's Hospital, Heilongjiang Province |
| 52 |  | Huanan County People's Hospital, Heilongjiang Province |
| 53 |  | Heilongjiang Jixi Mining Group General Hospital |
| 54 |  | Kedong County People's Hospital, Heilongjiang Province |
| 55 |  | Longjiang County People's Hospital, Heilongjiang Province |
| 56 |  | Mishan People's Hospital, Heilongjiang Province |
| 57 |  | People's Hospital of Nenjiang County, Heilongjiang Province |
| 58 |  | The First Hospital of Suihua City, Heilongjiang Province |
| 59 |  | People's Hospital of Tieli City, Heilongjiang Province |
| 60 | Hunan Province | The First People's Hospital of Changde City, Hunan Province |
| 61 |  | Cili County Traditional Chinese Medicine Hospital, Hunan Province |
| 62 |  | People's Hospital of Ningyuan County, Hunan Province |
| 63 |  | Taoyuan County People's Hospital, Hunan Province |
| 64 |  | Hunan Wangcheng District People's Hospital |
| 65 |  | The Fourth Hospital of Changsha City, Hunan Province |
| 66 | Jilin Province | Jilin Jiutai District Traditional Chinese Medicine Hospital |
| 67 | Liaoning Province | Central People's Hospital of Xiuyan Manchu Autonomous County, Liaoning Province |
| 68 |  | Zhangwu County People's Hospital, Liaoning Province |
| 69 | Inner Mongolia Autonomous Region | Ningcheng County Central Hospital, Inner Mongolia Autonomous Region |
| 70 |  | Tongliao Hospital, Inner Mongolia Autonomous Region |
| 71 |  | Yakeshi People's Hospital, Inner Mongolia Autonomous Region |
| 72 |  | Zhalantun People's Hospital, Inner Mongolia Autonomous Region |
| 73 | Shandong Province | Shandong Chengwu County People's Hospital |
| 74 |  | Ningjin County People's Hospital, Shandong Province |
| 75 |  | Pingdu People's Hospital, Shandong Province |
| 76 |  | The First People's Hospital of Tai'an City, Shandong Province |
| 77 |  | Xiajin County People's Hospital, Shandong Province |
| 78 |  | Shandong Zaozhuang Municipal Hospital |
| 79 | Shanxi Province | People's Hospital of Linyi County, Shanxi Province |
| 80 |  | Qinyuan County People's Hospital, Shanxi Province |
| 81 |  | Ruicheng County Traditional Chinese Medicine Hospital, Shanxi Province |
| 82 |  | Salt Lake District Branch of Shanxi Medical University First Hospital, Shanxi Province |
| 83 |  | Yangcheng County People's Hospital, Shanxi Province |
| 84 |  | Yicheng County Traditional Chinese Medicine Hospital, Shanxi Province |
| 85 |  | Yuanqu County People's Hospital, Shanxi Province |
| 86 |  | Shanxi Changzi County People's Hospital |
| 87 | Shaanxi Province | Baishui County Hospital, Shaanxi Province |
| 88 |  | Jingyang County Hospital, Shaanxi Province |
| 89 |  | Shaanxi Longxian People's Hospital |
| 90 |  | Meixian Traditional Chinese Medicine Hospital, Shaanxi Province |
| 91 |  | Shangnan County Hospital, Shaanxi Province |
| 92 |  | Yulin Second Hospital, Shaanxi Province |
| 93 |  | Ziyang County People's Hospital, Shaanxi Province |
